# Quantifying the relationship between increased disability and health care resource utilization, quality of life, work productivity, health care costs in patients with multiple sclerosis in the US

**DOI:** 10.1186/s12913-016-1532-1

**Published:** 2016-07-22

**Authors:** E. Jones, J. Pike, T. Marshall, X. Ye

**Affiliations:** Adelphi Real World, Adelphi Mill, Bollington, Macclesfield, Cheshire SK10 5JB UK; Abbvie Inc., 26525 Riverwoods Blvd., Mettawa, IL 60060 USA

**Keywords:** Disability, EDSS, Relapse, Health care utilization, Costs, Burden, Work productivity, Multiple sclerosis, Health-related quality of life

## Abstract

**Background:**

Multiple sclerosis (MS) is a chronic progressive condition affecting the central nervous system. Progression of MS results in increased level of disability and most patients will eventually experience some degree of functional impairment and impaired mobility. Costs and burdens escalate as MS disability increases. However, there is a lack of recent data on the impact of MS disability on the cost and burden among patients in the US.

**Methods:**

Data for this study were drawn from a real world, cross-sectional survey undertaken between 2013 and 2014. Neurologists completed detailed patient report forms (PRF) for the most recent consulting patients with MS (age >18 years). Patient’s perceptions of their diagnosis and health-related quality of life (HRQoL) were collected through a patient self-completion questionnaire (PSC). Regression analysis was used to evaluate the relationship between disability (determined by latest Expanded Disability Status Scale [EDSS] score) and current relapse and health care resource utilization, health care costs, HRQoL and work productivity.

**Results:**

PRF data were collected for 715 patients (335 also completed a PSC). Patients with higher disability scores (EDSS 3–5 and >5 vs <3 points) and current relapse (vs no current relapse) reported significantly greater health resource utilization for physician visits (*p* < 0.05) and hospitalizations (*p* < 0.05) in the preceding 12 months. In addition, they had poorer HRQoL (*p* < 0.05), were significantly more likely to be unemployed (*p* < 0.05) and to have had to stop working due to MS (*p* < 0.05). They also incurred significantly higher health care related costs, including costs for physician consultations, hospitalizations and therapy (*p* < 0.05). The total costs of care were $51,825, $57,889 and $67,116 for EDSS < 3, EDSS 3–5 and EDSS > 5 groups, respectively; $51,692 and $58,648 for non-relapse and relapse groups, respectively.

**Conclusions:**

For MS patients in the US, health resource utilization and healthcare care costs increase with progression of disability. As the disability worsens, patients also exhibit diminished HRQoL and lower work productivity. There is a need for treatments that slow down or delay disability progression among MS patients.

## Background

Multiple sclerosis (MS) is a chronic progressive condition affecting the central nervous system and characterized by localized areas of inflammation, demyelination and axonal degeneration [1]. This process results in a wide range of neurological symptoms, most commonly autonomic, visual, motor, and sensory in nature [1]. The progression of the neurological symptoms of MS may be ongoing (progressive MS) or associated with periodic worsening or relapses (relapsing-remitting MS) [2]. In the US, the number of MS patients has been estimated to be between 400,000 and 570,000 [3].

Progression of the neurological symptoms of MS results in increased level of disability and the majority of patients will eventually experience some degree of functional impairment and impaired mobility [4, 5]. In addition to impacting a patient’s ability to self-care and living independently [6], functional and mobility impairments can have an impact on the ability to work. Indeed, disease progression and the associated increase in disability have been associated with increased levels of unemployment [7, 8]. Symptoms of MS generally appear between the ages of 20 and 40 years, a time when individuals may be establishing their careers, starting a family or having a number of dependents to provide for [9]. The loss of independence and ability to work and provide for themselves and their families can have profound detrimental effects on patient’s health-related quality of life (HRQoL), social participation and psychological health, in addition to a considerable financial burden [10–13].

Several studies have suggested that costs and burdens associated with MS escalate with increased disability and may include the need for inpatient care in a skilled nursing facility for those most severely affected [14–17]. In addition, patients with MS accrue a considerable personal financial burden associated with missed work days, lost productivity and unemployment, as well as costs attributable to coping with the functional and mobility impairments including informal care costs, mobility aids, transportation costs, and home modifications [3, 18]. A limitation of the current evidence base regarding the burden and costs of MS is that the majority of the studies described above were conducted using data pre-2010 and as such a re-evaluation of the cost and burden of this chronic progressive condition is warranted. In particular, there is a need to examine the relationship between disability and health-care resource utilization (HCRU) and associated costs within the context of the new treatment options available, including oral disease-modifying drugs (DMDs), and the recognition that early intervention has the potential to slow the progression of the disease [19].

To address the lack of recent data on the disability associated with MS and its related impact on the burden on patients in the US, we conducted a real world, cross-sectional survey. The aim of the research was to quantify the relationship between increased disability and HRCU, healthcare cost, quality of life (QoL), ability to work and productivity while at work among patients with MS.

## Methods

### Study design

Data were drawn from the Adelphi MS III Disease Specific Programme (DSP® [20]). The Adelphi MS III DSP was a real-world, cross-sectional survey of neurologists caring for patients with MS in the USA. Physicians specializing in the management of patients with MS were identified using national databases, the internet, medical literature, and local physician networks and societies. A regional stratification approach was used to ensure geographical representativeness of the physician sample.

The survey, undertaken between 2013 and 2014, consisted of a physician-reported patient record form (PRF) and a patient self-completed questionnaire (PSC). The study population consisted of 715 patients with a diagnosis of MS who were >18 years of age and had been receiving treatment with any disease-modifying agent for the previous 12 months. Data for these patients were obtained from participating neurologists who completed a detailed PRF for the most recent consulting patients with a diagnosis of MS.

The research was conducted in full accordance with the US Health Insurance Portability and Accountability Act 1996 (HIPAA; www.hhs.gov/ocr/privacy/). All data were collected via ethically approved procedures through local fieldwork partners, including the informed consent of patients, and data was fully de-identified prior to receipt by Adelphi. Full details of the DSP methodology and of the methodology for the MS DSP have previously been published [20, 21].

### Physician-reported data

The PRF collected information from patient records on patient demographics and disease characteristics including time since diagnosis, symptoms at diagnosis, healthcare resource utilization (medication use, hospitalizations, emergency room [ER] visits, physician consultations, other healthcare professional [HCP] consultations, and supportive therapies), and whether the patient was considered to have experienced a recent relapse. Physicians were also asked to provide an Expanded Disability Status Scale (EDSS [22]) score where available. Only information available to the physician at the time of the patient consultation was collected.

### Patient-reported data

Patients for whom a PRF had been completed were invited to complete a PSC independently. The PSC collected information about the patient’s perceptions of their diagnosis, HRQoL (as assessed using the EQ-5D-3L health states and visual analog scale (VAS) and the Hamburg Quality of Life Questionnaire Multiple Sclerosis [HAQUAMS; [23]). The EQ-5D-3L consists of 5 health dimensions (mobility, self-care, usual activities, pain/discomfort and anxiety/depression) and each dimension is scored from no, some or extreme problems. These responses are used to derive an index score that ranges from −0.11 (worse than death) to 1 (full health), with lower scores representing lower quality of life. For the EQ-5D-3L, the country-specific value set for the USA was applied. The HAQUAMS consists of 38 items that examine general health perception and five health dimensions relevant for patients with MS (fatigue/thinking, mobility lower limb, mobility upper limb, social function and mood). A composite score is derived ranging from 1 to 5 with higher scores indicating poorer perceived HRQoL.

Patients were also asked to complete questions regarding their employment status, the impact of their MS on their ability to work and time lost to work as a result of their MS. Information of the work-related burden of MS was collected using the Work Productivity and Activity Impairment Questionnaire (WPAI) which was included as part of the PSC [24]. All answers were anonymised to preserve confidentiality and the patient responses were then matched to the corresponding PRF via patient and physician study numbers. The matching of these forms enabled analysis of corresponding data from the physician’s PRF.

### Statistical analysis

Analysis was restricted to RRMS patients receiving a DMD continuously for the previous 12 months.

The relationship between disability, as measured using the EDSS, and current relapse and healthcare resource utilization, healthcare costs, HRQoL, and work productivity was evaluated using regression analysis. Patient age, sex and body mass index were included as covariates in all regression analyses. These covariates were selected as being variables commonly regarded as potentially confounding or interacting with a variety of health states including MS. The clinical course of MS is age-related and older age tends to be associated with increased fragility and increased severity of any other comorbid health conditions. Gender has also been shown to impact the clinical expression of MS as well as responses to therapy. BMI may be indicative of current overall health. Regression analyses were chosen in order to strike a balance between the need for strong modelling and interpretability of the output data. The same approach was used for all analyses in order to ease the interpretation of coefficients. While using different models for some analyses may have provided a better fit to the model, it was felt that this would have offered little advantage and rendered interpretation more challenging.

The EDSS is a 10-point scale with 0.5 point increments reflecting increased levels of disability [22]. Scores of 1 to 4.5 define patients able to walk, scores of 5 to 9.5 define patients with impaired walking. Impairment in eight functional systems (pyramidal [limbs], cerebellar, brainstem, sensory, bowel and bladder, visual, cerebral and other) is reflected in increased EDSS scores. An EDSS score of <3 was defined as the reference group for comparison as these patients experience no, minimal or mild disability. Comparator groups were patients with EDSS score 3–5 who experience moderate to significant disability with limited impairment of activities and patients with EDSS scores >5 points who experience disability severe enough to impair daily activities and ability to work a full day. Patients reported to have suffered a relapse in the last 12 months were compared to the reference group of those not having suffered a relapse in the last 12 months, according to their physician.

Direct medical costs (health care practitioner consultations, hospitalizations, current treatment regimen) were calculated as US$/year. Given the lack of nationally representative healthcare costs for the US, unit costs were derived from a number of sources. The unit cost per hospital stay was based on the estimate reported by O’Brien et al. [25] adjusted for inflation to 2015 dollars; physician consultation costs were derived from the Annual Physician Fee Schedule Payment 2015 database [26] and medication costs were derived from the US Department of Veterans Affairs National Acquisition Center cost database [27]. Direct medical costs were calculated based on healthcare resource utilization as recorded in the PRFs for patients in all analysis cohorts.

Logistic regression was used for binary outcomes (producing odds ratios [ORs]), negative binomial regression for count outcomes (producing incidence rate ratios [IRRs]), and ordinary least squares for all other outcome types (producing coefficients). All regressions were adjusted for patient age, sex, and BMI. Standard errors were adjusted for possible correlation within reporting physician. All analyses were carried out using STATA 14.1 (StataCorp 2015. Stata Statistical Software: Release 14. College Station, TX: StataCorp LP).

## Results

A total of 109 neurologists provided PRFs for 715 patients with MS. Of these 715 patients, 335 also completed a PSC. No relevant demographic differences were noted between the cohort of patients providing a PSC and those who did not (data on file). The baseline demographic characteristics of the study population are shown in Table 1. In all, 31 % of the patient cohort was male, the mean age was 42.1 years and the mean time since diagnosis of MS was 6.9 years. Interferon beta-1a was the most common current DMDs (32.2 %) followed by Glatiramer acetate (24.9 %).

**Table 1:** Patient demographics and disease characteristics

	Patient population *N* = 715
Age, mean years (SD)	42.1 (10.7)
Male gender, N (%)	219 (30.6)
Ethnicity, N (%)	
White/Caucasian	552 (77.2)
Afro-Caribbean	98 (13.7)
Spanish/Hispanic	36 (5.0)
Other	25 (3.5)
Missing	4 (0.6)
Employment status, N (%)	
Employed	422 (59.0)
Housing status, N (%)	
Lives alone	139 (19.4)
Lives with partner/spouse	463 (64.8)
Lives with other friends/family	108 (15.1)
Missing	5 (0.7)
Relationship status, N (%)	
Single	112 (15.7)
In a relationship	476 (66.6)
Divorced/separated/widowed	111 (15.5)
Missing	16 (2.2)
Time since initial MS diagnosis, mean years(SD)	6.9 (5.3)^a^
Current DMDs, N (%)	
Interferon beta-1a	230 (32.2)
Interferon beta-1b	66 (9.2)
Glatiramer acetate	178 (24.9)
Dimethyl fumarate	93 (13.1)
Teriflunomide	22 (3.1)
Natalizumab	56 (7.8)

^a^Base = 594

*SD* standard deviation

### Association between MS disability and disease burden

#### Healthcare resource utilization

Compared to patients with an EDSS <3, those with higher disability scores made significantly more visits to a neurologist (EDSS 3–5, IRR 1.3; EDSS >5, IRR 1.4), MS nurse (EDSS 3–5, IRR 6.6; EDSS >5, IRR 44.4), a physiotherapist (EDSS 3–5, IRR 3.2; EDSS >5, IRR 9.9), and a urologist (EDSS 3–5, IRR 4.8; EDSS >5, IRR 7.2) in the previous 12 months (*p* < 0.05 for all comparisons; Table 2). Those in the highest disability group had significantly more hospitalizations in the previous 12 months (IRR 3.3, *p* < 0.05).

**Table 2:** Relationship between disability (EDSS) and relapse and health care resource utilization among patients with MS

Parameter, (95 % CI)	Disability (reference group EDSS <3; *n* = 411)		Relapsed (*n* = 303) (reference group no relapse; *n* = 341)	R^2^, %^b^
	EDSS 3–5 (*n* = 167)	EDSS > 5 (n = 66)		
Consultations in the last 12 months^a^				
Neurology	1.3* (1.2–1.5)	1.4* (1.2–1.6)	1.2* (1.1–1.4)	4.42
Primary care physician	1.0 (0.6–1.5)	0.9 (0.5–1.5)	1.5* (1.1–2.0)	0.71
MS specialist	1.0 (0.4–2.7)	0.5 (0.1–1.9)	2.4 (0.8–7.3)	1.84
MS Nurse	6.6* (2.4–18.2)	44.4* (8.0–248.2)	0.8 (0.3–2.1)	7.28
Internist	1.3 (0.5–3.2)	0.4 (0.1–1.3)	1.6 (0.6–4.2)	1.97
Emergency room doctor	1.9 (0.6–5.7)	0.8 (0.2–3.6)	15.6* (2.2–109.9)	12.98
Physiotherapist	3.2* (1.0–10.1)	9.9* (3.2–30.7)	1.5 (0.5–4.4)	5.06
Ophthalmologist	1.6 (0.9–3.0)	2.3 (1.0–5.4)	4.3* (2.0–8.9)	8.68
Urologist	4.8* (1.9–12.0)	7.2* (2.2–23.6)	1.8 (0.8–3.9)	7.67
Gastroenterologist	4.4 (0.6–32.3)	9.7 (0.9–101.9)	7.0* (1.2–41.4)	11.24
Psychiatrist	1.5 (0.4–5.4)	1.5 (0.3–7.1)	2.8* (1.0–7.7)	2.90
Other physician	3.9 (0.9–17.6)	6.3 (1.0–40.6)	1.8 (0.7–4.2)	7.69
Hospitalizations in the last 12 months^a^	1.7 (0.8–3.3)	3.3* (1.6–6.8)	7.1* (3.3–15.5)	15.37

*EDSS* Expanded Disability Status Scale, *MS* multiple sclerosis

*p < 0.05

^a^Negative binomial analysis to yield IRRs and 95 % CIs (covariates: patient age, sex and body mass index)

^b^McFadden’s R-Squared

Relapsing was notably associated with increased healthcare resource utilization (Table 2). Compared with patients with no relapse, those with relapse made significantly more visits to a neurologist, primary care physician, ER doctor, ophthalmologist, gastroenterologist or psychiatrist and had more hospitalizations in the previous 12 months (*p* < 0.05 for all; Table 2).

#### HRQoL

Patients with higher disability scores and disease relapse were more likely to perceive their MS as a major problem in their life that causes upset (*p* < 0.05 for EDSS 3–5 and EDSS >5 groups compared with EDSS <3 group; Table 3). Patients with greater disability were more likely to report poorer health status (EQ-5D) and poorer perceived QoL (EQ-5D VAS) (*p* < 0.05 for EDSS 3–5 and EDSS >5 groups compared with EDSS <3 group; Table 3).

**Table 3:** Relationship between disability (EDSS) and relapse and health-related quality of life among patients with MS

Parameter, (95 % CI)	Disability (reference group EDSS <3; n = 201)		Relapsed (*n* = 148) (reference group no relapse; *n* = 155)	R^2^, %
	EDSS 3–5 (*n* = 78)	EDSS >5 (*n* = 24)		
MS is a major problem in life and causes upset ^a,b^	1.6* (0.9–2.4)	3.1* (2.1–4.2)	1.0* (0.3–1.7)	26.79
EQ-5D^b,c^				
Health State	−0.09* (−0.13–-0.05)	−0.25* (−0.33–-0.18)	−0.05* (0.08–-0.01)	29.98
VAS	−11.1* (−14.8– -7.3)	−25.2* (−34.2– -16.2)	−2.9 (−5.9–-0.1)	31.93
HAQUAMS^b,d^				
Total score	0.5* (0.3–0.7)	1.1* (0.8–1.4)	0.2* (0.1–0.4)	39.95
Fatigue/thinking subscale	0.5* (0.2–0.7)	1.2* (0.8–1.6)	0.2 (−0.1–0.4)	27.51
Mobility/lower limb subscale	0.5* (0.3–0.7)	1.7* (1.3–2.1)	0.2* (0.1–0.4)	46.91
Mobility/upper limb subscale	0.5* (0.2–0.7)	1.3* (0.9–1.7)	0.3* (0.1–0.5)	36.45
Social function scale	0.3* (0.1–0.6)	0.4* (0.0–0.8)	0.2* (0.0–0.4)	11.99
Mood subscale	0.6* (0.4–0.9)	0.9* (0.6–1.3)	0.4* (0.2–0.6)	29.00

*EDSS* Expanded Disability Status Scale, *HAQUAMS* Hamburg Quality of Life Questionnaire Multiple Sclerosis, *MS* multiple sclerosis, *VAS* visual analog scale

*p < 0.05

^a^Ordinal scale of 1 to 10 (1 completely disagree to 10 completely agree)

^b^Ordinary least squares regression analysis to yield coefficients and 95 % CIs (covariates: patient age, sex and body mass index)

^c^Lower score indicates worse HRQoL

^d^Higher score indicates worse HRQoL

Using the MS-specific HRQoL tool HAQUAMS, increased disease severity was associated with an increased likelihood of worse HRQoL in terms of the overall score and scores on all subscales (*p* < 0.05 for EDSS 3–5 and EDSS >5 groups compared with EDSS <3 group).

Relapsing was associated with worse HRQoL measured by either EQ-5D or HAQUMAS (Table 3). In general, patients who experienced relapses had lower HRQoL than patients with no relapses (*p* < 0.05 for all except for EQ-VAS; Table 3).

#### Work and productivity

Compared to patients with an EDSS <3, those with a higher disability score were significantly more likely to be unemployed (EDSS of 3–5, OR 2.8; EDSS >5, OR 12.4; *p* < 0.05 for both comparisons) (Table 4). These patients were also significantly more likely to have been unable to get a job or a promotion due to their MS (EDSS 3–5 OR 4.8; EDSS >5 OR 17.0; *p* < 0.05 for both comparisons) or had to stop work altogether (EDSS 3–5 OR 2.6; EDSS >5 OR 10.4; *p* < 0.05 for both comparisons) due to their MS. Patients in the EDSS 3–5 group were significantly more likely to have experienced impairment while working (+13.3 %) and overall work impairment (+15.4 %) than those in the EDSS <3 group (*p* < 0.05 for both comparisons). Activity impairment was significantly greater for patients in both higher disability groups compared with those in the EDSS <3 group (EDSS 3–5, +16.8 %; EDSS >5, +33.6 %; *p* < 0.05 for both comparisons) as measured using the WPAI (Table 4).

**Table 4:** Relationship between disability (EDSS) and relapse and ability to work, productivity and employment status among patients with MS

Parameter, (95 % CI)	Disability (reference group EDSS <3; *n* = 201)		Relapsed (*n* = 148) (reference group no relapse; *n* = 155)	R^2^, %^c^
	EDSS 3–5 (*n* = 78)	EDSS >5 (*n* = 24)		
Unemployed^a^	2.8* (1.7–4.4)	12.4*(6.3–24.4)	1.1 (0.8–1.7)	15.20
Had to change job due to MS^a^	2.5 (0.7–8.5)	14.4* (4.5–46.0)	0.6 (0.2–1.7)	9.97
Had to reduce weekly working hours due to MS^a^	1.7 (0.8–3.5)	1.4 (0.4–4.6)	2.3* (1.3–4.1)	4.72
Unable to get a job or promotion due to MS^a^	4.8* (1.4–16.1)	17.0* (4.2–68.4)	1.7 (0.6–4.8)	17.51
Had to retire early due to MS^a^	1.1 (0.3–3.5)	2.2 (0.5–9.0)	1.9 (0.6–6.4)	13.22
Had to stop work altogether due to MS^a^	2.6* (1.1–6.3)	10.4* (3.5–30.8)	1.0 (0.4–2.1)	14.59
WPAI^b^				
% work time missed	4.2 (−2.6–11.0)	1.8 (−8.0–11.5)	3.2 (−1.2–7.7)	4.75
% impairment while working	13.3* (5.5–21.0)	12.0 (−26.5–50.6)	13.4* (6.3–20.6)	22.59
% overall work impairment	15.4* (6.7–24.1)	21.0 (−35.7–77.7)	12.8* (5.2–20.4)	24.02
% activity impairment	16.8* (9.3–24.2)	33.6*(23.2–44.0)	9.9* (3.3–16.5)	31.00

*EDSS* Expanded Disability Status Scale, *MS* multiple sclerosis, *WPAI* Work Productivity and Activity Impairment Questionnaire

*p < 0.05

^a^Logistic regression analysis to yield ORs and 95 % Cis (covariates: patient age, sex and body mass index)

^b^Ordinary least squares regression analysis to yield coefficients and 95 % CIs (covariates: patient age, sex and body mass index)

^c^R-Squared for ordinary least squares regression, McFadden’s R-Squared for logistic regression

Compared with patients without relapse, those with relapse were significantly more likely to have had to reduce their weekly working hours due to their MS (OR 2.3; *p* < 0.05) (Table 4).

### Economic costs associated with increased disability

Direct costs associated with health care practitioner consultations, hospitalizations and medications in the previous 12 months are shown in Table 5. As disability progressed, the direct costs associated with physician visits, hospitalization and medications also increased. The total costs of care were $51,825, $57,889 and $67,116 for EDSS < 3, EDSS 3–5 and EDSS > 5 groups, respectively. Relapse was also associated with increased costs. The total costs of care were $51,692 and $58,648 for the non-relapse and relapse groups, respectively.

**Table 5:** Direct health care costs (mean US$) over the previous 12 months among patients with MS by disability group (EDSS) and relapse status

Disability level	Mean (SD) US$			
	Physician visits	Hospitalization costs	Medication costs	Total direct costs
EDSS <3	1,659 (898.3)	1,546 (7017.1)	48,620 (10,612.2)	51,825 (12,741.8)
EDSS 3–5	2,545 (1,627.0)	6,217 (14402.5)	49,127 (12,319.3)	57,889 (20,020.7)
EDSS >5	2,491 (1,181.1)	14,303 (27,380.7)	50,321 (11,288.1)	67,116 (31,605.9)
No relapse	1,612 (986.6)	1,480 (7,464.0)	48,600 (10,520.9)	51,692 (13,017.6)
Relapsed	2,381 (1,347.2)	6,974 (17,250.4)	49,294 (11,808.4)	58,648 (22,128.2)

*EDSS* Expanded Disability Status Scale, *SD* standard deviation

Costs associated with health care practitioner consultations significantly increased among patients with higher disability scores compared with those with the lowest disability scores (EDSS 3–5, +$692; EDSS >5, +$730; p < 0.05) (Table 6). Higher levels of disability were associated with higher costs over the previous 12 months for hospitalizations (+$10,883; *p* < 0.05) and total MS costs (consultations, hospitalizations and cost of treatment) (+$13,324; *p* < 0.05; Table 6).

**Table 6:** Relationship between disability (EDSS) and relapse and direct health care costs over the previous 12 months among patients with MS

Parameter, (95 % CI) ^a^	Disability (reference group EDSS <3)		Relapse (reference group no relapse)	R^2^, %
	EDSS 3–5	EDSS >5		
Costs of consultations in last 12 months (US$)	692* (242.6–1141.3)	730* (358.1–1101.4)	464* (232.4–695.9)	16.53
Cost of hospitalisations in last 12 months (US$)	3,007 (−363.4–6377.6)	10,883* (4025.6–17739.8)	3,693* (1313.9–6072.3)	10.84
Cost of current treatment regimen in last 12 months (US$)	318 (−2129.6–2765.6)	1,711 (−1466.3–4888.3)	233 (−1704.6–2170.2)	1.55
Total costs (US$)	4,017 (−916.5–8950.7)	13,324* (4872.1–21774.8)	4,390* (1248.3–7531.8)	8.75

*EDSS* Expanded Disability Status Scale, *MS* multiple sclerosis

*p < 0.05

^a^Ordinary least squares regression analysis to yield coefficients and 95 % CIs (covariates: patient age, sex and body mass index)

Relapsing was notably associated with increased health care costs (Table 6). Compared with those with no relapse, patients with relapse accrued significantly higher costs over the previous 12 months for physician consultations (+$464; *p* < 0.05), hospitalizations (+$3693; *p* < 0.05) and total MS costs (+$4390; *p* < 0.05).

## Discussion

This study has highlighted the considerable burden of disability associated with MS on patients in the US using the real-world survey data from MS patients and neurologists caring for these patients.

Although previous studies have demonstrated that MS is associated with considerable rates of unemployment [28, 29] and that absenteeism and presenteeism are common among patients with MS [30], large-scale evaluations of the real-world association between MS and employment and productivity in the workplace are lacking. In 915 US-based patients with MS receiving treatment in 2013, increased disability was associated with a marked impact on the ability of patients to work, as reflected by a greater likelihood of being unemployed, a greater likelihood of having to reduce their working hours or to have changed their job as a result of their condition. In a cohort of 337 individuals with the relapsing-remitting form of MS, 14 % of patients reported having to take time away from work due to their MS (absenteeism) and 47 % reported lost productivity at work as a result of their MS (presenteeism) [30]. Recently, several studies in small cohorts of patients with MS have suggested that fatigue, cognitive function and motor function may mediate the impact of disability on work status [31–33]. Our results confirm and extend these previous findings and highlight the relevance of increased disability as a driver of decrements in the capacity to work among patients with MS.

In a cohort of 1510 patients receiving treatment for MS in the US, adherence to DMDs was shown to be associated with significantly lower healthcare resource utilization [34]. However, this analysis did not include an evaluation of the degree of disability for these patients. Our research has shown that, increased disability and current relapse were both associated with an increase in health care resource utilization. This was reflected in the increased numbers of health care practitioner visits, notably to neurologists, MS nurses and physiotherapist. The likelihood of requiring at least one hospitalization in the previous 12 months was also significantly increased for the most disabled patients compared with those in the least disabled category. Of particular note was the observation that as disability increased, the likelihood of needing to visit the ER increased. Indeed, for patients with an EDSS score >5 the number of ER visits was 15 times higher than that for patients with an EDSS score <3. Recently, Oynhausen et al. examined the acute care needs of patients with MS presenting to the Mount Sinai ER [35]. They found that patients with MS were most likely to present in the ER with non-neurological problems such as urinary tract infections.

Perhaps unsurprisingly given the pervasive nature of the impact of MS on all aspects of the daily lives of patients with MS, increased disability was associated with an increased likelihood of reduced HRQoL. A previous study among patients with MS covering the period 1998 to 2009 demonstrated the negative impact of MS on HRQoL and reported that patients with MS lost 10.4 quality-adjusted life years as a consequence of their disease compared to individuals without MS [3]. Among patients with the relapsing-remitting MS, physical symptoms such as pain, stiffness and muscle spasms were related to physical QoL [36]. The presence of depression and cognitive impairments were more closely associated with work impairments. Our results confirm these findings and highlight the association between disability and HRQoL in MS patients. The specific drivers of HRQoL decrements in MS are not well defined. A recent study among 97 patients identified increased EDSS score and the presence of depressive symptomatology as relevant determinant of QoL among patients with MS [37]. Consistent with this, a larger study in a cohort of 949 adults with MS in Canada found that increased disability, depression and anxiety, fatigue and physical comorbidity were all associated with decreased HRQoL [38]. Together, these studies support the importance of targeting disability in order to achieve improved HRQoL for patients.

In addition to detrimental effects of increased disability on work capacity and productivity, our results also demonstrated an association between increaseddisability and the ability of patients to undertake activities of daily living. As MS-related disability increased, the likelihood of patients to be able to successfully self-care and complete usual household tasks reduced, and the need for informal care and support increased. An increase in disability was associated with an increased likelihood of requirement assistance with a range of usual self-care activities from getting out of bed to preparing meals and travelling outside the home. This may be because the EDSS was used to describe disability in the current research, a tool that focuses mainly on functional capabilities, specifically walking ability, to determine disability. A previous study showed that cognitive aspects of the disease, such as the ability of the patient to make decisions about daily tasks, were associated with caregiver employment [39]. A systematic evaluation of the impact of the symptom profile of patients with MS on caregiver burden is lacking and may inform the holistic support patients with MS and their informal caregivers require.

The burden of and costs associated with MS have been investigated using many different models including cross-sectional studies, retrospective analyses, patient self-completion questionnaires and data obtained from healthcare claims databases. Such studies have shown the costs associated with MS disability to be considerable for individuals, their families and for society as a whole [3, 18, 40, 41]. Initiation of DMDs early in the disease course has been shown to slow the neurodegenerative changes that may underpin physical disability and to reduce relapse frequency [42]. Our research has demonstrated that such a strategy could offer considerable benefits both in terms of the associated cost burden and the personal burden to patients, allowing patients to continue to work, care for themselves and their families. Our research has demonstrated the association between disability and relapses and direct medical costs; specifically, elevated health care professional consultations, and, increased hospitalization costs for those with the highest levels of disability.

When considering the results presented here, it is important to acknowledge the limitations of the design of this research. Cross-sectional analyses such as presented here are hypothesis-generating in that they allow identification of associations (rather than causal relationships) between variable factors and the outcomes of interest. Data were derived by a survey-based methodology with physicians providing data available at the time of consultation. The pragmatic approach to accruing the patient sample, while practical in terms of deriving a large patient population, may have excluded relevant types of patients including those not currently receiving treatment or those not currently requiring physician consultation. Our patient cohort consisted of predominantly Caucasian, female patients consistent with the epidemiology of MS. Despite this, our cohort may not be entirely representative of the US MS patient population and, as it consisted of only patients consulting their physician at the time the research was conducted. Although pragmatic, this approach may have limited the generalizability of our observations to the entire MS population in the US. An additional limitation of this research that must be acknowledged is the lack of nationally representative cost data for the US. Actual patient-level cost data is determined by the healthcare plan to which the patient belongs. As it was beyond the scope of the current research to collect actual patient-level cost data, alternative unit costs sources were utilized. Where possible, official cost data from national social insurance programmes administered by the US federal government were utilized as these were regarded as the most reliable and nationally representative sources. A disadvantage of these sources is that they provide average costs regardless of patient-level diagnosis and so may not necessarily represent the actual costs of care for patients with MS. Despite these limitations, the aim of the research was to evaluate the impact of disease progression as reflected by increased disability measured using the EDSS, as such our patient cohort was stratified by EDSS score, with a score <3 group providing an internal control for comparisons.

## Conclusions

In summary, our research has shown that the burden and cost of MS increases with progression of disability as measured by the EDSS and with current relapse. Our research highlights the need for treatments targeted at slowing down or improving the physical disabilities associated with MS to improve patient work capacity, HRQoL, and patient ability to self-care as well as to mitigate increased health resource utilization and its associated costs.

## Abbreviations

DMD, disease-modifying drug; DSP, disease specific programme; EDSS, expanded disability status scale; ER, emergency room; HCP, healthcare professional; HCRU, Health-Care Resource Utilization; HIPAA, Health Insurance Portability and Accountability Act 1996; HRQoL, health-related quality of life; MS, multiple sclerosis; PRF, Patient Record Form; PSC, patient self-completion questionnaire; QoL, quality of life; US, United States; VAS, visual analog scale; HAQUAMS, Hamburg quality of life questionnaire multiple sclerosis; WPAI, work productivity and activity impairment questionnaire; RRMS, relapse remitting multiple sclerosis

## References

[1] Compston A, Coles A (2008). Multiple sclerosis. Lancet.

[2] Lublin FD, Reingold SC (1996). Defining the clinical course of multiple sclerosis: results of an international survey. Neurology.

[3] Campbell JD, Ghushchyan V, McQueen RB (2014). Burden of multiple sclerosis on direct, indirect costs and quality of life: National US estimates. Mult Scler Relat Disord.

[4] Noseworthy J, Lucchinetti C, Rodriguez M, Weinshenker BG (2000). Multiple sclerosis. N Engl J Med.

[5] Souza A, Kelleher A, Cooper R, Cooper RA, Iezzoni LI, Collins DM (2010). Multiple sclerosis and mobility-related assistive technology: systematic review of the literature. J Rehabil Res Dev.

[6] Salter AR, Cutter GR, Tyry T, Marrie RA, Vollmer T (2010). Impact of loss of mobility on instrumental activities of daily living and socioeconomic status in patients with MS. Curr Med Res Opin.

[7] Edgely K, Sullivan M, Dehoux E (1991). A survey of multiple sclerosis: II. Determinants of employment status. Can J Rehabil.

[8] Simmons RD, Tribe KL, McDonald EA (2010). Living with multiple sclerosis: longitudinal changes in employment and the importance of symptom management. J Neurol.

[9] Milo R, Kahana E (2010). Multiple sclerosis: geoepidemiology, genetics and the environment. Autoimmune Rev.

[10] Kwiatkowlski A, Marissal JP, Pouyfaucon M, Vermersch P, Hautecoeur P, Dervaux B (2014). Social participation in patients with multiple sclerosis: correlations between disability and economic burden. BMC Neurol.

[11] Murray ED, Buttner EA, Price BH, Daroff R, Fenichel G, Jankovic J, Mazziotta J (2012). Depression and Psychosis in Neurological Practice. Bradley’s neurology in clinical practice (6^th^ edn).

[12] Sutliff MH (2010). Contribution of impaired mobility to patient burden in multiple sclerosis. Curr Med Res Opin.

[13] Zwibel HL (2009). Contribution of impaired mobility and general symptoms to the burden of multiple sclerosis. Adv Ther.

[14] Cassado V, Martinez-Yelamos S, Martinez-Yelamos A, Carmona O, Alonso L, Romero L (2006). Direct and indirect costs of multiple sclerosis in Baix Llobregat (Catalonia, Spain), according to disability. BMC Health Serv Res.

[15] Ertekin O, Ozakbas S, Idiman E (2014). Caregiver burden, quality of life and walking ability in different disability levels of multiple sclerosis. NeuroRehabilitation.

[16] Noyes K, Bajorska A, Weinstock-Guttman B, Mukamel DB (2013). Is there extra cost of institutional care for MS patients?. Mult Scler Int.

[17] Parise H, Laliberite F, Lefebvre P, Duh MS, Kim E, Agashivala N (2013). Direct and indirect cost burden associated with multiple sclerosis relapses: excess costs of persons with MS and their spouse caregivers. J Neurol Sci.

[18] Kobelt G, Berg J, Lindgren P, Refdrikson S, Jonsson B (2006). Costs and quality of life of patients with multiple sclerosis in Europe. J Neurol Neurosurg Psychiatry.

[19] Noyes K, Bajorska A, Chappel A (2011). Cost-effectiveness of disease-modifying therapy for multiple sclerosis: a population-based study. Neurology.

[20] Anderson P, Benford M, Harris N, Karavali M, Piercy J (2008). Real-world physician and patient behavior across countries: Disease-Specific Programmes – a means to understand. Curr Med Res Opin.

[21] Pike J, Jones E, Rajagopalan K, Piercy J, Anderson P (2012). Social and economic burden of walking and mobility problems in multiple sclerosis. BMC Neurol.

[22] Kurtzke JF (1983). Rating neurologic impairment in multiple sclerosis: an expanded disability status scale (EDSS). Neurology.

[23] Gold SM, Heesen C, Schultz H, Guder U, Monch A, Gbadamosi J (2001). Disease specific quality of life instruments in multiple sclerosis: validation of the Hamburg Quality of Life Questionnaire in Multiple Sclerosis (HAQUAMS). Mult Scler.

[24] Zhang W, Bansback N, Boonen A, Young A, Singh A, Anis AH (2010). Validity of the work productivity and activity impairment questionnaire – general health version in patients with rheumatoid arthritis. Arthritis Res Ther.

[25] O’Brien JA, Ward AJ, Patrick AR, Caro J (2003). Cost of managing an episode of relapse in multiple sclerosis in the United States. BMC Health Serv Res.

[26] Annual Physician Fee Schedule Payment, Medicare. Centers for Medicare & Medicaid Services. Available at: https://www.cms.gov/Medicare/Medicare-Fee-for-Service-Payment/PhysicianFeeSched/index.html?redirect=/PhysicianFeeSched/. Accessed September 2015.

[27] US Veterans Affairs. National Acquisitions Center. Available at: http://www1.va.gov/nac/index.cfm?cboContractNumbers=&cboContractorName=&txtCriteria1=TECFIDERA+240MG+CAP+%3A%3A+DIMETHYL+FUMARATE+240MG+CAP+EC&TxtNDC=&txtPackage=&cboVAClass=&Sort=1&search=Search&template=Search_Pharmaceutical_Catalog. Accessed September 2015.

[28] Julian LJ, Vella L, Vollmer T, Hadjimichael O, Mohr DC (2008). Employment in multiple sclerosis. Exiting and re-entering the work force. J Neurol.

[29] Roessler RT, Rumrill PD (2003). Multiple sclerosis and employment barriers: a systematic perspective on diagnosis and intervention. Work.

[30] Glanz BI, Degano IR, Rintell DJ, Chitnis T, Weiner HL, Healy BC (2012). Work productivity in relapsing multiple sclerosis: associations with disability, depression, fatigue, anxiety, cognition, and health-related quality of life. Value Health.

[31] Baughman BC, Basso MR, Sinclair RR (2015). Staying on the job: The relationship between work performance and cognition in individuals diagnosed with multiple sclerosis. J Clin Exp Neuropsychol.

[32] Cadden M, Arnett P (2015). Factors associated with employment status in individuals with multiple sclerosis. Int J MS Care.

[33] Coyne KS, Boscoe AN, Currie BM (2015). Understanding drivers of emplyment changes in a multiple sclerosis population. Int J MS Care.

[34] Yermakov S, Davis M, Calnan M (2015). Impact of increasing adherence to disease-modifying therapies on healthcare resource utilization and direct medical and indirect work loss costs for patients with multiple sclerosis. J Med Econ.

[35] Oynhausen S, Alcauskas M, Hannigan C, Bencosme Y, Muller M, Lublin F (2014). Emergency medical care of multiple sclerosis patients: primary data from the Mount Sinai Resource Utilization in Multiple Sclerosis project. J Clin Neurol.

[36] Williams AE, Vietri JT, Isherwood G, Flor A (2014). Symptoms and association with health outcomes in relapsing-remitting multiple sclerosis: Results of US patient survey. Mult Scler Int.

[37] Fernandez-Jimenez E, Arnett PA (2014). Impact of neurological impairment, depression, cognitive function and coping on quality of life of people with multiple sclerosis: A relative importance analysis. Mult Scler.

[38] Berrigan LI, Fisk JD, Patten SB et al. Health-related quality of life in multiple sclerosis: Direct and indirect effects of comorbidity. Neurology 2016;Epub March 9. 10.1212/WNL.0000000000002564 PMC4831037 26962068

[39] Buchanan RJ, Huang C, Zheng Z (2013). Factors affecting employment among informal caregivers assisting people with multiple sclerosis. Int J MS Care.

[40] Carroll CA, Fairman KA, Lage MJ (2014). Updated cost-of-care estimates for commercially insured patients with multiple sclerosis: retrospective observational analysis of medical and pharmacy claims data. BMC Health Serv Res.

[41] O’Connell K, Kelly SB, Fogarty E, Duggan M, Buckley L, Hutchinson M (2014). Economic costs associated with an MS relapse. Mult Scler Relat Disord.

[42] Faber RS, Sand IK (2015). Optimizing the initial choice and timing of therapy in relapsing-remitting multiple sclerosis. Ther Adv Neurol Disord.

